# Are YouTube Videos Sufficient for Educational Purposes for Robotic Right Hemicolectomy Learning and Has Complete Mesocolic Excision Changed That?

**DOI:** 10.5152/tjg.2023.22827

**Published:** 2023-12-01

**Authors:** Ali Bal, Muhammet Kadri Çolakoğlu, Volkan Öter, Erol Pişkin, Erdal Birol Bostancı

**Affiliations:** 1 Department of Surgical Oncology, University of Health Sciences Faculty of Medicine, Ankara City Hospital, Ankara, Turkey; 2 Department of Gastroenterology Surgery, University of Health Sciences Faculty of Medicine, Ankara City Hospital, Ankara, Turkey

**Keywords:** Robotic right hemicolectomy, educational purposes, complete mesocolic excision, YouTube video platform

## Abstract

**Background/Aims:**

The aim of this study is to evaluate the efficiency for educational purposes by evaluating the videos published on YouTube channel, which is an open source video sharing platform, for robotic right hemicolectomy procedure.

**Materials and Methods:**

: We searched YouTube website to choose video clips that included information about robotic right hemicolectomy for right colon cancer. All videos were analyzed according to the criteria like quality of videos, quality of teaching, and modified Laparoscopic Surgery Video Educational Guidelines.

**Results:**

There were 16 complete mesocolic excision and 56 noncomplete mesocolic excision videos in the study. According to the Likert scale, calculated complete mesocolic excision scores were analyzed better than the noncomplete mesocolic excision group and this difference was statistically significant (*P < .*0001). The teaching quality scores of complete mesocolic excision videos were higher than noncomplete mesocolic excision group and this result was statistically significant (*P = .*02). The videos were scored according to the modified Laparoscopic Surgery Video Educational Guideline, and the score difference was statistically significant between complete mesocolic excision and noncomplete mesocolic excision videos (*P < .*001). The video power index was higher (mean 5.52 ± 15.56 vs. mean 1.66 ± 3.41) in the complete mesocolic excision group, but there was no statistically significant difference between the 2 groups (*P = .*086).

**Conclusions:**

: Most of the robotic right hemicolectomy videos on the YouTube platform are insufficient in terms of educational capacities. Complete mesocolic excision-containing videos are slightly superior in this respect to noncomplete mesocolic excision videos, as considering a new technique can make video presenters more attentive. In our opinion, if the images presented to the video platforms are to be used for educational purposes, they must undergo a certain evaluation and screening process.

**Main Points**

- Surgery is still the most successful treatment method for right colon cancer.
- Laparoscopic and robotic surgery are current treatment methods in minimally invasive surgery.
- In robotic right hemicolectomy for right colon cancer, complete mesocolic excision-containing videos are slightly superior to noncomplete mesocolic excision videos for educational purposes.

## Introduction

Surgery is still the most successful treatment method for right colon cancer. Since the first successful right hemicolectomy operation performed by Reybard in 1832, the surgical technique described has been refined and minimally invasive methods have gained importance in the treatment of right colon cancer in today’s modern age.^1^ Minimally invasive approaches, which started and developed primarily with laparoscopic methods, gradually began to leave their place to the period of robotic surgery.

The traditional surgical training model has historically been based on the “watch first, perform later” model.^2^ Although studies on cadaveric surgical procedures and animal models have somewhat reversed this, it does not seem possible for all surgical students to receive these trainings, and these procedures are limited to training-specific or new procedures only. In the age of technology, surgical simulation models are very useful in closing this gap, and nowadays surgeons can also find the opportunity to perform operations in simulation models first, instead of just watching them.^3^ However, the high cost of these new technological models limits their accessibility. All these factors bring us back to the very beginning, the watch first, perform later model. Because in the era of social media, it is now very easy to access videos of many interventional processes and it has become possible even from mobile phones that we carry with us all the time. Video-based training has been shown to be an effective and useful method in surgical training,^4^ and this method is quite accessible and inexpensive. However, it is a question that wonders whether there will be a difference between watching the operation with a master who describes and explains all the stages of the procedure to us and watching a video processed with various add-and-remove methods on the screen. Especially if these videos are watched from a platform that everyone can access and have different sources, it is a big problem how sufficient these videos can be for your surgical training.

Although an answer to this question has been sought for many laparoscopic methods, studies in the field of robotic surgery are limited.^5-8^ The aim of this study is to evaluate the efficiency for educational purposes by evaluating the videos published on YouTube channel, which is an open source video sharing platform, for robotic right hemicolectomy procedure. While doing this, the quality of the videos, the quality of teaching, and the items of the Laparoscopic Surgery Video Educational Guideline (LAP-VEGaS) were evaluated together. In addition, the popularity of these videos was evaluated using the Video Power Index (VPI), and the results were presented in detail.

## Materials and Methods

Ethics committee approval and informed consent were not required because it was a retrospective video comparison study on an open share video platform.

We searched YouTube website (www.youtube.com) on February 17, 2022, to choose video clips that included information about robotic right hemicolectomy for right colon cancer. The term “robotic right hemicolectomy” and associated terms were used to search and found the related video clips. After all videos were evaluated, we used the similar inclusion and exclusion criteria for the videos to be selected, as in our previous study^9^ and they were determined as follows;

Inclusion criteria:

1. More than 100 views.
2. Performing robotic right colon surgery.
3. Videos with writing or narration in English language.

Exclusion criteria:

1. Open, hand-assisted or laparoscopic surgery.
2. Single port videos.
3. Colon surgery for patient with situs inversus.
4. Surgical videos for non-malignant masses or diseases.
5. Videos with additional organ resections.

After filtering the videos based on inclusion and exclusion criteria, all videos were evaluated separately by 2 experienced surgeons interested in robotic surgery and analyzed according to the methods presented below. After the evaluation is completed by each, the videos are evaluated together and in case of any disagreement, an agreed decision had been achieved with consensus.

With the prevalence of complete mesocolic excision (CME) surgery in right colon cancer, the evaluated videos were analyzed independently by dividing them into CME and non-CME groups to see the effects of a new method among YouTube viewers. When the evaluation of all the selected videos was finished, all the results were sorted according to 4 setting categories. In these categories, the method used in our previous study on the subject was also performed.^9^

In the first category, the demographic features of the videos were examined and scored. These features were the time from the day the video was uploaded until February 17, 2021, the total number of views of the video, the daily average number of views of the video, the number of likes of the video, the number of dislikes of the video, the number of comments written for the video, audio commentary, subtitles commentary, and video capture quality (dpi). Video scores were categorized into 4 groups using the modified Likert scale method by reviewer surgeons, as poor (0-3), moderate (4-6), good (7-8), or very good (9-10) in terms of their contribution to the surgical education.

In the second category, the teaching quality of the videos was assessed. These features were examined by scoring the indispensable steps of the surgery one by one. A minimally invasive right hemicolectomy technique can be described in its simplest form in 5 steps. These are (1) mobilization of the right colon, (2) mobilization of the transverse colon, (3) control of mesentary, (4) anastomosis, and (5) closure and re-inspection. The steps we use described for robotic right hemicolectomy were exposure of the ileocecal junction area, illustration of the origin of ileocolic artery and vein ligation, dissection of apical lymph nodes and illustration of ileocolic origin, exposure of the duodenum, illustration of the terminally ileum transection, illustration of the hepatic flexura mobilization, illustration of the resection of the transverse colon, illustration of the middle colic artery dissection, demonstration of the anastomosis, and illustration of Toldt’s fascia dissection. Also operative procedure as lateral to medial, medial to lateral, top to down, or bottom to top is recorded.^10^ The education quality of the evaluated video was scored by giving “1” point to each of these steps.

Laparoscopic Surgery Video Educational Guidelines have been created recently to evaluate the quality of educational videos.^11, 12^ The purpose of this guide is to establish a standard by evaluating videos from patient presentation to operation details and video quality. In the third category, all videos were scored according to the whole (37 criteria) LAP-VEGaS guideline and after that videos were scored according to modified LAP-VEGaS criteria including only major 15 criteria that were created by 2 experienced surgeons from the LAP-VEGaS criteria which were selected for suitable for making evaluation in YouTube videos. Modified and essence LAP-VEGaS were scored as “0” point if not visible and “1” point if the video showed a complete critical view of safety.

In the fourth category, VPI was calculated to evaluate the popularity of the videos. We use the VPI to assess both the view and the like ratio of the videos. The VPI was calculated as follows: first, calculate the like ratio (like number × 100)/(like number + dislike number) and the view ratio (number of views/days); then, the VPI is equal to the like ratio × view ratio/100.^13^

### Statistical Analysis

Data analysis was performed with Statistical Package for the Social Sciences Statistics version 22.0 (IBM Corp.; Armonk, NY, USA). Descriptive statistics was presented as frequencies (n) and percentages (%) for categorical variables and mean or median (SD, range) for continuous and ordinal variables. Mann–Whitney *U* test was performed for comparing median values, and the mean differences were evaluated by Student’s *t*-test. The chi-square test was performed to examine the relationship between 2 categorical variables. A *P* < .05 was considered statistically significant.

## Results

The related term “robotic right hemicolectomy” was searched on YouTube on the given date and a total number of 119 videos were evaluated. After 47 videos were extracted using the inclusion and exclusion criteria, 72 videos remained and these videos were included in the study. At first, the videos in the study were divided into 2 groups group I: CME group and group II: non-CME group. There were 16 complete mesocolic excision and 56 non-CME videos in the study. After the results of the whole group were presented, the groups with and without CME were evaluated within themselves and possible differences were analyzed.

In the first category, the demographic features of the videos were examined in total and each group; the median time from the day the video was uploaded until February 17, 2021, was 606 days (range min-max; 27-1915 days) and the median length of duration for the videos was 535 seconds (range min-max; 112-22 561 seconds). Other demographic characteristics of the videos are presented in Table 1 for the whole and each group. According to these demographic characteristics and the watchability of the video, the Likert scale was used and evaluated subjectively between the 2 audience surgeons, and the results of the 2 groups were compared. Among the videos, there was no video that could be evaluated as “poor” in CME group but 15 (26.8%) in the non-CME group. While only 4 videos (5.5%) were classified as “very good” in the total group, all of the videos were in the CME group. Videos classified as “good” were in the majority, both in total and within each group. When evaluated according to the Likert scale, calculated CME scores were analyzed better than the non-CME group and this difference was statistically significant (*P < .*0001).

For the second category, the teaching qualities of the videos are shown in Table 2. Scoring was made based on whether the 10 operation steps, which were determined to be indispensable for robotic right hemicolectomy, were presented in the video. A score of “1” was given for the presence of each step. When the whole group is evaluated, it was seen that less than half of the group (32 videos, 44.4%) scored 9-10. While this rate was 62.5% (10 videos) in the CME group, it was 39.2% (22 videos) in the non-CME group. This result indicates that more than half of the videos do not show at least 1 of the important steps of surgery and therefore most of them have low teaching capacity. Table 2 shows each of these steps and whether they are presented or not, in both groups. After the calculation of total scores, it was observed that the teaching quality scores of CME videos were higher than non-CME group and this result was statistically significant (*P = .*02) (mean value of Group I: 9.75 ± 1.23; mean value of Group II: 8.46 ± 2.04).

For the third category, the videos were scored according to LAP-VEGaS guidelines. The positivity average ratio of these 37 criteria was 35.32% and the negativity average ratio of these 37 criteria was 64.68%. For the reason that most of these 37 parameters were not included in the videos, only 15 parameters were selected that were considered necessary. The modified LAP-VEGaS criteria (major 15 criteria) were selected from the LAP-VEGaS criteria (37 criteria). These criteria were scored and shown in Table 3. Based on these 15 parameters, 27 videos scored 0-5 points, 28 videos scored 6-10 points, and only 17 videos scored 11-15 points. These results indicate that most of the videos do not meet the criteria considering the modified LAP-VEGas criteria. When the groups were analyzed separately, it is seen that only 9 (56.2%) videos in the CME group and 8 videos (15.3%) in the non-CME group received 11-15 points. When the 2 groups were compared, it was found that the score difference was statistically significant between CME and non-CME videos (*P < .*001).

The VPI was calculated to evaluate the popularity of the videos. The median VPI value was 0.77 (range 0.05-63) and the mean ratio was 2.51 ± 7.92. When the popularity of CME and non-CME videos was evaluated based on the VPI score, it was observed that the median score of the group I was higher (mean 5.52 ± 15.56 vs. mean 1.66 ± 3.41), while there was no statistically significant difference between the 2 groups (*P = .*086). The *P* values of this result and other statistical calculations are presented together in Table 4.

## Discussion

Since the beginning of 2000s, social media has become an indispensable part of our daily lives. In particular, the intense interest in video-sharing platforms has paved the way for the use of these platforms for educational purposes. Among these educational platforms, medical education has also found a place for itself in a short time and social media has started to be used intensively among medical students.^14^ Simultaneously, the surgical videos that surgical specialists publish on these platforms for various purposes have also started to be used as a training tool for both surgical assistants and surgical specialists. However, the use of these uncontrolled and unsupervised surgical videos for educational purposes has become the most fundamental problem and the literature has begun to seek answers in all areas of surgery. The most important question raised in this regard is the educational capacities of the videos on social media platforms. The general surgery literature did not fail to question the usability of these videos in terms of education, and the use of various procedures on video platforms was tried to be questioned.^15-20^

It has been shown that the frequency of social media use among general surgeons is high and one of the most frequently used platforms for educational purposes is YouTube.^21^ Due to the widespread use of laparoscopy and the possibility of recording videos, the majority of the videos presented on YouTube are laparoscopic procedures. For this reason, laparoscopic procedures constitute the majority of the videos whose educational capacity is questioned, but the publications in which robotic procedures are questioned for this purpose are relatively few.^22, 23^ In addition, the effect of new techniques on this education capacity has never been mentioned in the literature before. For all these reasons, in this study, the educational capacity of robotic right hemicolectomy procedures published on YouTube and the impact of the CME technique on the education capacity of the need to explain a new technique were tried to be analyzed.

For this purpose, the videos included in the study were evaluated in 4 different categories based on the model in a similar study we performed before.^9^ First, the demographic features of the videos were analyzed and 2 surgeons were asked to score these videos using the Likert scale, based on these features. The Likert scale is a subjective assessment that may vary depending on the person, and although it does not reflect the general, it can give an idea about the evaluated subject. In this study, the impression of the videos on the surgeon at the first viewing was evaluated using this scale and the majority of the videos were classified as good. However, this evaluation was made by evaluating the video quality or features such as narration and subtitles and is not for education capacities. The main conclusion that should be interpreted in the results is that CME videos are better evaluated for these features than non-CME videos for both surgeons. This may indicate that video publishers are more careful when interpreting a new technique. When evaluated in terms of teaching capacities, our results are not different from other surgical results in the literature. Considering the main steps of the surgical procedures presented, it has been shown that most of the videos presented on YouTube do not include one or more steps. However, as with the demographic characteristics of the videos, this capacity was found to be higher in the CME group than in the non-CME group. This confirms our first assertion. The LAP-VEGaS criteria were set forth for this purpose and determined the steps that should be included in educational videos. These criteria are mostly on educational schematics and the scope of surgical steps is limited. In our opinion, it is not sufficient alone in the evaluation of training videos.^9^ Therefore, it is only 1 of the 4 steps used in the videos evaluated in our study. In our study, the majority of the videos do not include the steps of LAP-VEGaS criteria, just like the teaching capacity. However, the CME group was again superior to the non-CME group in this respect. Video Power Index, on the other hand, is a scale that reveals the popularity of videos by taking various parameters into account. Considering this scale, there was no difference between the CME and non-CME groups in terms of popularity. This result shows that no matter how different the educational characteristics are, video viewers cannot reach this distinction and can watch videos that may be insufficient at the same rate.

This issue has been evaluated in 2 previous studies in the literature, but laparoscopic videos were also included in the evaluation in both studies. Uzunoglu et al^22^ analyzed both laparoscopic and robotic right hemicolectomy videos that they included in the study and did not find the educational value of most of them sufficient. Similarly, Moctezuma-Velázquez et al^24^ reported that the educational quality in the laparoscopic right hemicolectomy videos they evaluated was not sufficient. Although the number of videos included in the study is limited, our study has shown that the educational capacity of the robotic right hemicolectomy videos on the YouTube video-sharing platform is not sufficient.

In conclusion, most of the robotic right hemicolectomy videos on the YouTube video platform are insufficient in terms of educational capacities. Complete mesocolic excision-containing videos are slightly superior in this respect to non-CME videos, as considering a new technique can make video presenters more attentive. In our opinion, if the images presented to the video platforms are to be used for educational purposes, they must undergo a certain evaluation and screening process.

## Figures and Tables

**Table 2.** The Assessment of the Teaching Quality of the Videos

	Group I (CME) n = 16 Videos	Group II (non-CME) n = 56 Videos
Exposure of the ileocecal junction area		
Yes	8 (50%)	25 (44.6%)
No	8 (50%)	31 (55.4%)
Illustration of the origin of ileocolic artery and vein ligation		
No	0	9 (16.1%)
Yes	16 (100%)	47 (83.9%)
Dissection of apical lymph nodes		
No	0	5 (8.9%)
Yes	16 (100%)	51
Exposure to the duodenum		
No	0	3 (5.4%)
Yes	16 (100%)	53 (94.6%)
Illustration of the terminal ileum transection		
No	2 (12.5%)	14 (25%)
Yes	14 (87.5%)	42 (75%)
Illustration of the hepatic flexura mobilization		
No	3 (18.8%)	14 (25%)
Yes	13 (81.2%)	42 (75%)
Illustration of the middle colic artery dissection		
No	0	33 (58.9%)
Yes	16 (100%)	23 (41.1%)
Illustration of the resection of the transverse colon		
No	3 (18.8%)	12 (21.4%)
Yes	13 (81.2%)	44 (78.6%)
Illustration of Toldt’s fascia dissection		
No	3 (18.8%)	14 (25%)
Yes	13 (81.2%)	42 (75%)
Demonstration of the anastomosis		
Yes, intracorporeal	12 (75%)	43 (76.8%)
Yes, extracorporeal	2 (12.5%)	6 (10.7%)
No	2 (12.5%)	7 (12.5%)

**Table 3.** The Evaluation of the Modified Laparoscopic Surgery Video Educational Guideline Criteria

	Group I (CME) n = 16 Videos	Group II (non-CME) n = 56 Videos
1. Pathology and procedure title		
No	4 (25%)	23 (41.1%)
Yes	12 (75%)	33 (58.9%)
2. Author information		
No	3 (18.8%)	22 (39.3%)
Yes	13 (81.2%)	34 (60.7%)
Case presentation		
1. Patient anonymity		
No	0	0
Yes	16 (100%)	56 (100%)
2. Imaging		
No	3 (18.8%)	50 (89.3%)
Yes	13 (81.2%)	6 (10.7%)
3. Basic patient characteristics		
No	6 (37.5%)	45 (80.4%)
Yes	10 (62.5%)	11 (10.6%)
4. Pre-op examinations and treatments		
No	6 (37.5%)	44 (78.6%)
Yes	10 (62.5%)	12 (21.4%)
Procedure		
1. Room layout and necessary equipment		
No	3 (18.8%)	36 (64.3%)
Yes	13 (81.2%)	20 (35.7%)
2. Position of the operating team and patient		41 (73.2%)
No	6 (37.5%)	15 (26.8%)
Yes	10 (62.5%)	
3. Position of the trocars on the patient		31 (55.4%)
No	2 (12.5%)	25 (44.6%)
Yes	14 (87.5%)	
4. Anatomical demonstration		16 (28.6%)
No	3 (18.8%)	40 (71.4%)
Yes	13 (81.2%)	
5. Step-by-step procedure		
No	3 (18.8%)	20 (35.7%)
Yes	13 (81.2%)	36 (64.3%)
Results		
1. Results of the procedure, operative time, blood loss, cosmetics with a picture of healed wounds, length of hospital stay, and postoperative morbidity		
No	9 (56.3%)	50 (89.3%)
Yes	7 (43.7%)	6 (10.7%)
2. Specimen photos and histopathological examination report		
No	7 (43.7%)	46 (82.1%)
Yes	9 (56.3%)	10 (17.9%)
Educational content		
1. Pictures, snapshots, diagrams, and tables		
No	5 (31.2%)	45 (80.4%)
Yes	11 (68.8%)	11 (10.6%)
2. Voiced/written comments		
No	0	0
Yes	16 (100%)	56 (100%)

**Table 4.** The Evaluation of the Teaching Quality, Modified Laparoscopic Surgery Video Educational Guideline Criteria Likert Scale, and VPI of the Videos According to Academic and Individual Videos

Total Number of Videos n = 72	Group I n = 16 Videos	Group II n = 56 Videos	*P*
Teaching quality of the videos (number of patients)			
0%-25% (0-3 points)	0	3	*P = .*02
265-50% (4-6 points)	1	8	
51%-75% (7-8 points)	5	23	
76%-100% (9-10 points)	10	22	
Modified LAP-VEGaS			
0-5 points	2	25	*P < .*001
6-10 points	5	23	
11-15 points	9	8	
According to the Likert scale			
Poor (0-3)	0	15	*P < .*0001
Moderate (4-6)	3	15	
Good (7-8)	9	26	
Very good (9-10)	4	0	
Mean video power index (range minimum–maximum)	5.52 ± 15.56	1.66 ± 3.41	*P = .*086

LAP-VEGaS, Laparoscopic Surgery Video Educational Guideline.

**Table 1.** Demographic Features of the Videos

	Group I (CME) n = 16 Videos	Group II (Non-CME) n = 56 Videos	All Videos n = 72 Videos
Median number of views of the videos	358 (125-2884)	552 (112-22 561)	535.5 (112-22 561)
Median number of daily average number of views	1 (0-5)	1 (0-26)	1 (0-26)
Median number of likes of the videos	4 (0-16)	4 (0-429)	4 (0-429)
Mean number of dislikes of the videos	0.31 ± 0.70	0.64 ± 1.17	0.54 ± 0.96
Mean number of comments	0.19 ± 0.75	0.93 ± 3.28	0.76 ± 2.9
Number of audio commentaries	11 (68.8%)	23 (41%)	34 (47.2%)
Number of subtitles commentary	8 (50%)	27 (48.2%)	35 (48.6%)
Number of video capture quality (dpi)			
240	0	3 (5.4%)	3 (4.16%)
360	0	2 (3.6%)	2 (2.77%)
480	5 (31.2%)	19 (33.9%)	24 (33.3%)
720	11 (68.8%)	32 (57.1%)	43 (59.7%)
Modified Likert ratio			
Poor	0	15 (26.8%)	15 (20.8%)
Moderate	3 (18.8%)	15 (26.8%)	28 (38.8%)
Good	9 (56.2%)	26 (46.4%)	35 (48.6%)
Very good	4 (25%)	0	4 (5.5%)
